# Association Between Participation in Clinical Trials and Overall Survival Among Children With Intermediate- or High-risk Neuroblastoma

**DOI:** 10.1001/jamanetworkopen.2021.16248

**Published:** 2021-07-08

**Authors:** Skye Balyasny, Sang Mee Lee, Ami V. Desai, Samuel L. Volchenboum, Arlene Naranjo, Julie R. Park, Wendy B. London, Susan L. Cohn, Mark A. Applebaum

**Affiliations:** 1 College of the Liberal Arts, Penn State University, University Park, Pennsylvania; 2 Department of Public Health Sciences, University of Chicago, Chicago, Illinois; 3 Department of Pediatrics, University of Chicago, Chicago, Illinois; 4 Children’s Oncology Group Statistics and Data Center, Department of Biostatistics, University of Florida, Gainesville; 5 Seattle Children’s Hospital, University of Washington, Seattle; 6 Boston Children’s Hospital and Dana-Farber Cancer Institute, Harvard Medical School, Boston, Massachusetts

## Abstract

**Importance:**

Participants in clinical trials may experience benefits associated with new therapeutic strategies as well as tight adherence to best supportive care practices.

**Objectives:**

To investigate whether participation in a clinical trial is associated with improved survival among children with neuroblastoma and investigate potential recruitment bias of patients in clinical trials.

**Design, Setting, and Participants:**

This cohort study included pediatric patients with intermediate- or high-risk neuroblastoma in North American studies who were included in the International Neuroblastoma Risk Group Data Commons and who received a diagnosis between January 1, 1991, and March 1, 2020.

**Exposure:**

Enrollment in a clinical trial.

**Main Outcomes and Measures:**

Event-free survival and overall survival (OS) of patients with intermediate- or high-risk neuroblastoma enrolled in an up-front Children’s Oncology Group (COG) clinical trial vs a biology study alone were analyzed using log-rank tests and Cox proportional hazards regression models. The racial/ethnic composition and the demographic characteristics of the patients in both groups were compared.

**Results:**

The cohort included 3058 children with intermediate-risk neuroblastoma (1533 boys [50.1%]; mean [SD] age, 10.7 [14.7] months) and 6029 children with high-risk neuroblastoma (3493 boys [57.9%]; mean [SD] age, 45.8 [37.4] months) who were enrolled in a Children’s Oncology Group or legacy group neuroblastoma biology study between 1991 and 2020. A total of 1513 patients with intermediate-risk neuroblastoma (49.5%) and 2473 patients with high-risk neuroblastoma (41.0%) were also enrolled in a clinical trial, for a cohort total of 3986 of 9087 children (43.9%) enrolled in a clinical trial. The prevalence of prognostic markers for the clinical trial and non–clinical trial cohorts differed, although representation of patients from racial/ethnic minority groups was similar in both cohorts. Among patients with intermediate-risk neuroblastoma, OS was higher among those who participated in a clinical trial compared with those enrolled only in a biology study (OS, 95% [95% CI, 94%-96%] vs 91% [95% CI, 89%-94%]; *P* = .01). Among patients with high-risk neuroblastoma, participation in a clinical trial was not associated with OS (OS, 38% [95% CI, 35%-41%] in the clinical trial group vs 41% [95% CI, 38%-44%] in the biology study group; *P* = .23).

**Conclusions and Relevance:**

Approximately 44% of patients in this large cohort of patients with neuroblastoma were enrolled in up-front clinical trials. Compared with children not enrolled in clinical trials, a higher prevalence of favorable prognostic markers was identified among patients with intermediate-risk neuroblastoma enrolled in clinical trials, and unfavorable features were more prevalent among patients with high-risk neuroblastoma enrolled in clinical trials. No evidence of recruitment bias according to race/ethnicity was observed. Participation in a clinical trial was not associated with OS in this cohort, likely reflecting the common practice of treating nontrial participants with therapeutic and supportive care regimens used in a previous therapeutic trial.

## Key Points

**Question:**

Is enrollment in a clinical trial associated with improved survival among children with neuroblastoma?

**Findings:**

This cohort study of 9087 children with neuroblastoma identified a higher prevalence of favorable prognostic markers among children with intermediate-risk neuroblastoma who were enrolled in clinical trials and more unfavorable features among children with high-risk neuroblastoma compared with risk-matched patients not enrolled in a clinical trial. Overall survival was superior for children with intermediate-risk neuroblastoma who were enrolled in a clinical trial but not children with high-risk neuroblastoma who were enrolled in a clinical trial.

**Meaning:**

This study suggests that participaton in a clinical trial was not associated with survival in this high-risk cohort, likely reflecting the practice of treating nontrial participants with regimens used in a previous therapeutic trial.

## Teaser

This cohort study investigates whether participation in a clinical trial is associated with improved survival among children with neuroblastoma and investigates potential recruitment bias of patients in clinical trials.

## Introduction

Therapeutic clinical trials have enabled the development of new approaches that have improved the survival of patients with cancer.^1,2,3,4^ Although patients receiving experimental regimens in therapeutic clinical trials may experience benefits associated with new treatment strategies, those randomly assigned to receive the standard of care may also experience benefits associated with strict adherence to treatment schedules, dosing, and supportive care required by study protocols. A single-institution study demonstrated that children with cancer treated in clinical trials showed a trend toward improved outcomes.^5^ We therefore hypothesized that children with neuroblastoma would also experience benefits associated with clinical trial enrollment.

Throughout the world, treatment of neuroblastoma is tailored according to the risk of relapse and death based on a combination of clinical and genetic prognostic biomarkers.^6^ In the Children’s Oncology Group (COG), patients with low-risk disease have excellent outcomes, and current studies are evaluating whether subsets of these children may be cured with observation alone (NCT02176967). A series of COG and legacy North American cooperative groups (Pediatric Oncology Group [POG] and Children’s Cancer Group [CCG]) studies have established that patients with intermediate-risk disease also have excellent outcomes with surgery and moderate-dose chemotherapy.^6^ Successive studies (POG 9243,^7^ COG A3961,^8^ and COG ANBL0531^9^) have demonstrated that therapy reduction approaches effectively maintain excellent outcomes for these patients. Similar results have been observed in European protocols for low-risk and intermediate-risk neuroblastoma.^10,11,12^

For patients with high-risk disease, successive randomized COG (CCG 3891, COG A3973, and ANBL0532) and European clinical trials testing increasingly intensive, multimodality treatments have led to new standards of care and improved survival.^6,13,14,15,16,17^ Despite these successes, participation in an unproven therapeutic trial carries risk, and the experimental nature of trials may cause anxiety for patients and families.^18^ Although a substantially larger proportion of pediatric oncology patients are enrolled in clinical trials compared with adults with cancer,^19^ more than half of all patients with neuroblastoma are not treated in a clinical trial owing to many factors, including family and clinician preference and receiving a diagnosis when no open trial is available. For these patients, treatment is generally based on the regimen demonstrating the best outcome in the most recently completed clinical trial.

The International Neuroblastoma Risk Group (INRG) Data Commons includes clinical phenotype, tumor biology, and outcome data on patients enrolled in COG (ANBL00B1) or legacy (9047) biology studies. Since 2000, the ANBL00B1 biology study has served as the infrastructure for rapid and reliable acquisition of tumor prognostic markers for risk classification and enrollment in COG clinical trials.^20^ Approximately 500 to 600 patients per year are enrolled in this biology study, representing 70% to 80% of all patients with neuroblastoma diagnosed in North America.^20^ Clinical trial registration numbers for the subset of patients with neuroblastoma enrolled in up-front COG or legacy North American cooperative group clinical trials are also available in the INRG Data Commons. To investigate the potential benefit associated with participating in a clinical trial, we compared the outcome of 3986 patients with intermediate- or high-risk neuroblastoma in the INRG Data Commons who were enrolled in a clinical trial with the outcome of 5101 patients not enrolled in a trial but treated with standard of care. Because patient selection bias is known to affect the outcome of a trial, we assessed the clinical features and tumor biomarkers of the cohort enrolled in a clinical trial vs a biology study only. Potential racial/ethnic disparities in trial participation were also evaluated.

## Methods

### Patients and Variables

In this cohort study, data from patients with intermediate- or high-risk neuroblastoma in the INRG Data Commons were assessed.^20^ The study cohort included patients who received a diagnosis between January 1, 1991, and March 1, 2020. Survival analyses were conducted among the subset of patients with known outcomes who received a diagnosis prior to January 1, 2017, to ensure at least 3 years of follow-up. Patients were evaluated according to enrollment in a cooperative biology study (POG 9047 or COG ANBL00B1, which centrally collected patient and tumor data and outcomes) but not an up-front clinical trial vs those enrolled in a risk-based (CCG, POG, or COG) clinical trial for patients with a new diagnosis. Because only COG and POG also collected data from patients enrolled in a biology trial but not an up-front clinical trial, the study cohort was limited to patients in North America. Patient data abstracted from the INRG Data Commons included age at diagnosis, sex, race, ethnicity, International Neuroblastoma Staging System (INSS) stage, year of diagnosis, *MYCN* (GenBank 4613) amplification status, ploidy, grade of differentiation, histologic characteristics, aberrations of 1p and 11q, and the mitosis-karyorrhexis index (MKI). The INRG Data Commons and data use are approved by the University of Chicago institutional review board, which waived consent as all data were deidentified. This study followed the reporting requirements of the Strengthening the Reporting of Observational Studies in Epidemiology (STROBE) reporting guideline.

Risk group was assigned according to the 2006 COG classification system^21^ using INSS stage, age, histologic characteristics, ploidy, and *MYCN* status. As the classification system changed over time, all patients were assigned a risk group based on features available in the INRG Data Commons and analyzed accordingly. Thus, all comparisons were between patients meeting identical criteria for risk assignment. Because outcome data for half of the patients enrolled in ANBL0532 were not available in the INRG Data Commons, these patients were excluded from survival analyses.

### Statistical Analysis

The χ^2^ test and the Wilcoxon rank sum test compared characteristics of patients according to clinical trial enrollment status. Event-free survival (EFS) and overall survival (OS) were estimated by Kaplan-Meier methods, and the differences between groups were evaluated using the log-rank test.^22^ Point estimates of EFS and OS were calculated at 10 years from diagnosis because patients treated in older trials were often lost to follow-up after this time.^23,24^ In addition, we conducted univariate and multivariate analyses of established prognostic markers (age, INSS stage, *MYCN* amplification status, histologic characteristics, and ploidy) within subsets of patients with intermediate- or high-risk disease included in estimates of EFS and OS using Cox proportional hazards regression models.^25^ In multivariable models, we adjusted potentially confounded factors with outcomes among patients’ characteristics significant at *P* < .05 in univariate analysis. Factors were dropped if more than 20% of patients had missing data. The proportional hazards assumption was validated for all models. To assess the association of changes in standard of care resulting from successive clinical trials, the OS of patients with high-risk disease who were not treated in an up-front clinical trial was analyzed over time. Statistical analyses were performed using Stata, version 16 (StataCorp LLC) and R, version 3.6.0 (R Group for Statistical Computing). All *P* values were from 2-sided tests, and results were deemed statistically significant at *P* < .05.

## Results

### Cohort Characteristics

There were 3058 patients with intermediate-risk neuroblastoma (1533 boys [50.1%]; mean [SD] age, 10.7 [14.7] months) and 6029 patients with high-risk neuroblastoma (3493 boys [57.9%]; mean [SD] age, 45.8 [37.4] months) in the final analytic cohort (Table 1). We identified 14 723 patients who received a diagnosis between 1991 and 2020 and were treated at COG, POG, or CCG institutions. Patients with low-risk neuroblastoma (n = 4791) were excluded. In addition, we excluded 845 patients for whom risk group assignment could not be determined owing to unknown stage (n = 263), unknown *MYCN* status (n = 497), or unknown histologic characteristics (n = 85) for those older than 18 months with *MYCN*-nonamplified, INSS stage 3 tumors. Between 1991 and 2000, a median of 399 patients (interquartile range [IQR], 327-410 patients) were enrolled in the POG 9047 biology study per year. After activation of the COG biology study (ANBL00B1) in 2001, a median of 561 patients (IQR, 542-629 patients) were enrolled each year between 2001 and 2019. In the United States and Canada, approximately 700 to 800 new cases of neuroblastoma are diagnosed annually.^26,27^ Thus, approximately 50% to 57% of all patients with neuroblastoma who received a diagnosis in the 1990s and 70% to 80% of patients who received a diagnosis since 2001 are included in the INRG Data Commons. Of the 3058 patients with intermediate-risk disease, 41 (1.3%) were enrolled in a high-risk clinical trial and were excluded from survival analyses. Similarly, 68 patients with high-risk disease (1.1%), 56 with *MYCN*-amplified tumors, enrolled only in an intermediate-risk trial and were excluded from survival analyses.

**Table 1.** Characteristics of Patients With High- or Intermediate-risk Neuroblastoma

Feature	High risk, No. (%)^a^		*P* value	Intermediate risk, No. (%)^a^		*P* value
	Biology trial only (n = 3556)	Clinical trial (n = 2473)		Biology trial only (n = 1545)	Clinical trial (n = 1513)	
Age at diagnosis, median (IQR), mo	34.7 (21.6-54.1)	36.9 (24-54.3)	.002	7.8 (3.4-12.4)	6.7 (2.7-11.5)	.002
Age at diagnosis, d						
<547	603 (17)	324 (13.1)	<.001	1374 (88.9)	1348 (89.1)	.80
≥547	2953 (83)	2149 (86.9)		171 (11.1)	165 (10.9)	
Sex						
Male	2062 (58)	1431 (57.9)	.91	762 (49.3)	771 (51)	.63
Female	1492 (42)	1042 (42.1)		783 (50.7)	742 (49)	
Unknown	2	0		0	0	
Race						
White	2529 (80.8)	1742 (80.8)	.41	1118 (84.4)	1151 (86.5)	.07
Black	446 (14.2)	316 (14.7)		135 (10.2)	132 (9.9)	
Native American	21 (0.7)	7 (0.4)		9 (0.7)	9 (0.7)	
Asian	124 (4)	84 (3.9)		54 (4)	36 (2.7)	
Hawaiian or Alaska native	12 (0.3)	5 (0.2)		9 (0.7)	2 (0.2)	
Unknown	424	319		220	183	
Ethnicity						
Non-Hispanic	2560 (88)	1868 (88.7)	.42	1103 (85.6)	1054 (87.8)	.10
Hispanic	349 (12)	237 (11.3)		186 (14.4)	146 (12.2)	
Unknown	647	368		256	313	
INSS stage						
4	2945 (83.9)	2151 (88.6)	<.001	547 (35.8)	435 (29)	<.001
4s	54 (1.5)	17 (0.7)		229 (15)	269 (18)	
3	469 (13.4)	244 (10.1)		732 (48)	572 (38.2)	
2	44 (1.2)	15 (0.6)		19 (1.2)	223 (14.8)	
Unknown	44	46		18	14	
Lactate dehydrogenase level, U/L						
<900	477 (41.8)	166 (43.8)	.50	413 (79.1)	252 (63.3)	<.001
≥900	664 (58.2)	213 (56.2)		109 (20.9)	146 (36.7)	
Unknown	2415	2094		1023	1115	
Serum ferritin level, ng/mL						
<90	212 (23.1)	70 (24.2)	.69	225 (57.5)	133 (50.2)	.06
≥90	707 (76.9)	219 (75.8)		166 (42.5)	132 (49.8)	
Unknown	2637	2184		1154	1248	
Time of diagnosis						
1991-1999	558 (15.7)	792 (32)	<.001	205 (13.3)	521 (34.4)	<.001
2000-2008	1290 (36.3)	651 (26.3)		468 (30.3)	602 (39.8)	
2009-2016	1264 (35.5)	777 (31.4)		645 (41.7)	346 (22.9)	
2017-2020	444 (12.5)	253 (10.3)		227 (14.7)	44 (2.9)	
*MYCN*						
Nonamplified	1728 (55.7)	1194 (57.3)	.26	1488 (100)	1477 (100)	NA
Amplified	1375 (44.3)	891 (42.7)		0	0	
Unknown	453	388		57	36	
INPC						
Favorable	181 (6.9)	85 (4.7)	.002	1157 (94.4)	1154 (92.2)	.02
Unfavorable	2429 (93.1)	1734 (95.3)		68 (5.6)	98 (7.8)	
Unknown	946	654		320	261	
Ploidy						
Hyperdiploid	1308 (49.1)	680 (45.8)	.04	925 (74.6)	912 (78.1)	.04
Hypodiploid or diploid	1357 (50.9)	806 (54.2)		315 (25.4)	256 (21.9)	
Unknown	891	987		305	345	
Tumor diagnosis						
Neuroblastoma	2262 (89.6)	1395 (88.9)	.48	1149 (90.9)	943 (95.2)	<.001
Ganglioneuroblastoma or ganglioneuroma	264 (10.4)	175 (11.1)		115 (9.1)	48 (4.8)	
Unknown	1030	903		281	522	
Grade of differentiation						
Undifferentiated or poorly differentiated	2247 (95.7)	1697 (97.7)	.001	937 (90)	904 (90.9)	.52
Differentiating	100 (4.3)	40 (2.3)		104 (10)	91 (9.1)	
Unknown	1209	736		504	518	
MKI						
Low	661 (31.6)	508 (32.4)	.79	655 (65)	715 (72.7)	<.001
Intermediate	595 (28.4)	452 (28.8)		282 (28)	230 (23.4)	
High	836 (40)	610 (38.8)		70 (7)	38 (3.9)	
Unknown	1464	903		538	530	
Aberration at 1p						
Absent	378 (59.2)	361 (55.2)	.15	251 (89.3)	474 (88.6)	.75
Present	261 (40.8)	293 (44.8)		30 (10.7)	61 (11.4)	
Unknown	2917	1819		1264	978	
Aberration at 11q						
Absent	444 (69.9)	439 (69)	.73	245 (87.8)	478 (91.1)	.15
Present	191 (30.1)	197 (31)		34 (12.2)	47 (8.9)	
Unknown	2921	1837		1266	988	
Gain of 17q						
Absent	0	44 (48.4)	NA	0	43 (91.5)	NA
Present	0	47 (51.6)		0	4 (8.5)	
Unknown	3556	2382		1545	1513	

Abbreviations: INPC, International Neuroblastoma Pathology Classification; INSS, International Neuroblastoma Staging System; IQR, interquartile range; MKI, mitosis-karyorrhexis index; NA, not applicable.

SI conversion factors: To convert lactate dehydrogenase to microkatals per liter, multiply by 0.0167; ferritin to micrograms per liter, multiply by 1.0.

^a^ Percentages calculated from nonmissing data.

### Characteristics of Patients With Intermediate-risk Disease

Of the 3058 patients with intermediate-risk disease, 1513 (49.5%) were enrolled in an up-front clinical trial, and 1545 were enrolled in biology trials only (Table 1). A total of 132 of 1330 patients enrolled in a clinical trial (9.9%) and 135 of 1325 patients enrolled in a biology study (10.2%) were Black. Hispanic patients made up 13.3% (332 of 2489) of the group of patients with intermediate-risk disease. Compared with patients enrolled only in a biology study, those enrolled in an intermediate-risk clinical trial were more likely to have favorable risk features, including non–stage 4 disease (1064 of 1499 [71.0%] vs 980 of 1527 [64.2%]; *P* < .001) and tumors with low MKI (715 of 984 [72.7%] vs 655 of 1007 [65.0%]; *P* < .001) and/or hyperdiploid (912 of 1168 [78.1%] vs 925 of 1240 [74.6%]; *P* = .04). Conversely, patients in clinical trials were less likely than those in biology studies to have favorable histologic characteristics (1154 of 1252 [92.2%] vs 1157 of 1225 [94.4%]; *P* = .02). There were no differences according to age (>18 months), sex, race/ethnicity, or *MYCN* amplification.

### Outcomes for Patients With Intermediate-risk Disease

**Figure 1.**
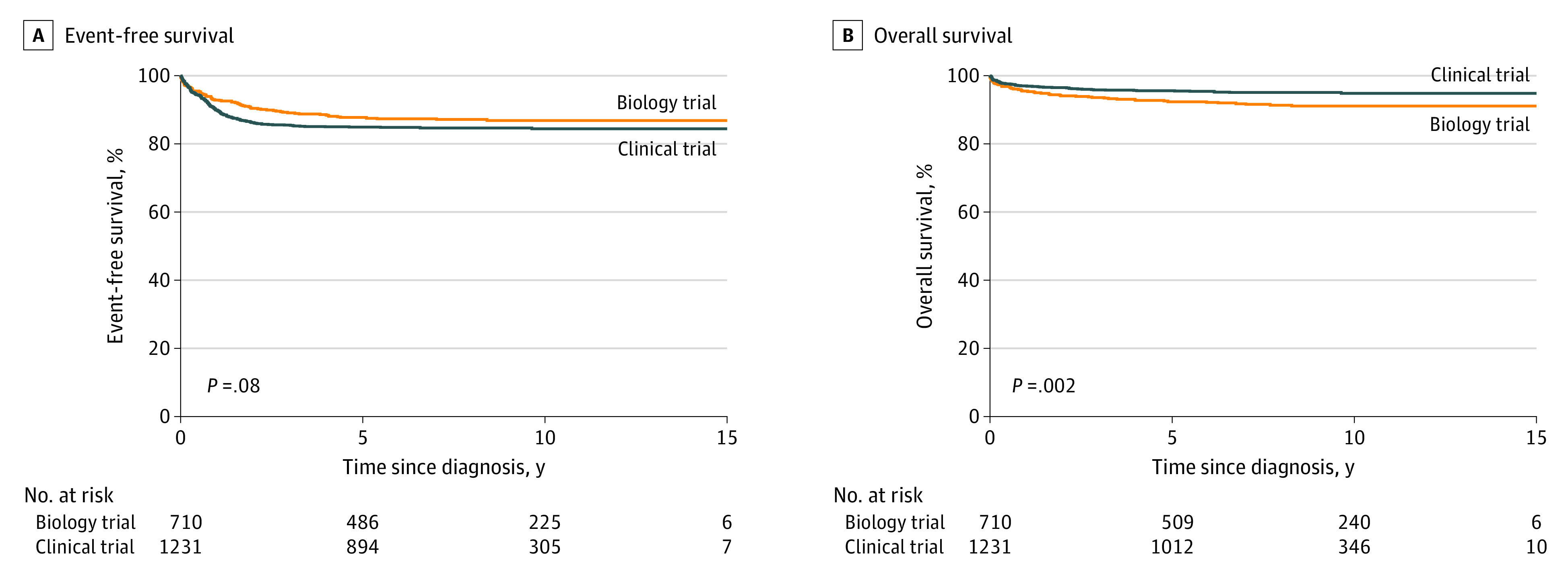
Outcomes for Patients With Intermediate-risk Neuroblastoma A, Probability of event-free survival. B, Probability of overall survival. Patients were enrolled in 4 clinical trials (Pediatric Oncology Group 9243, Children’s Cancer Group 3881, Children’s Oncology Group [COG] A3961, or COG ANBL0531 [n = 1231]) or in a biology study alone (n = 710).

To assess differences in outcomes according to enrollment in a clinical trial vs biology study alone, we focused on studies COG A3961, ANBL0531, CCG 3881, and POG 9243, each of which enrolled more than 100 patients. No difference in EFS was observed between patients enrolled in a clinical trial (n = 1231) between 1991 and 2011 (excluding 2006 because no studies were open that year) compared with those enrolled in a biology study alone in those same years (n = 710) (85% [95% CI, 83%-87%] vs 87% [95% CI, 84%-90%] at 10 years; *P* = .08) (Figure 1A). The median follow-up time of survivors was 8.5 years (IQR, 6.2-10.6 years) for patients enrolled in a clinical trial and 8.4 years (5.2-10.6 years) for those enrolled in a biology study. A Cox proportional hazards regression model showed no difference in the hazard ratio (HR) for EFS according to clinical trial enrollment (HR, 1.36; 95% CI, 0.97-1.92; *P* = .07) when accounting for stage, histologic characteristics, and ploidy (eTables 1 and 2 in the Supplement). Overall survival was significantly higher for patients with intermediate-risk disease who were enrolled in a clinical trial than for those enrolled in a biology study (95% [95% CI, 94%-96%] vs 91% [95% CI, 89%-93%]; *P* = .002) (Figure 1B and Table 2). However, in a multivariable model accounting for age, disease stage, and ploidy, enrollment in a clinical trial vs a biology study did not retain a statistically significantly higher OS (HR, 0.68; 95% CI, 0.45-1.03; *P* = .07) (eTables 1 and 2 in the Supplement).

**Table 2.** EFS and OS Among Patients Enrolled in Clinical Trials vs Biology Studies Alone According to Diagnostic Era and Risk Assignment

Trial	Clinical trial			Biology study only^a^			*P* value	
	No.	10-y EFS (95% CI), %	10-y OS (95% CI), %	No.	10-y EFS (95% CI), %	10-y OS (95% CI), %		
							EFS	OS
High-risk disease								
CCG 3891	505	22 (18-26)	27 (23-31)	243	28 (22-34)	30 (24-36)	.02	.93
COG A3973	417	43 (38-48)	49 (45-53)	564	42 (38-46)	47 (43-51)	.52	.38
Intermediate-risk disease								
CCG 3881	220	86 (81-91)	96 (93-99)	75	92 (86-98)	95 (90-99)	.17	.42
POG 9243	162	81 (76-86)	93 (90-96)	88	86 (79-93)	92 (88-96)	.19	.85
A3961	452	87 (83-91)	96 (93-99)	335	86 (82-90)	91 (88-94)	.99	.01
ANBL0531	397	84 (80-88)	95 (92-98)	269	88 (84-92)	92 (88-96)	.10	.20

Abbreviations: CCG, Children’s Cancer Group; COG, Children’s Oncology Group; EFS, event-free survival; OS, overall survival; POG, Pediatric Oncology Group.

^a^ Biology study–only patients were those who were not enrolled in a clinical trial but received a diagnosis in the years matching those of the clinical trial (CCG 3891: 1991-1996, COG A3973: 2001-2005, CCG 3881: 1991-1995, POG 9243: 1992-1996, COG A3961: 1997-2005, and COG ANBL1531: 2007-2011).

### Characteristics of Patients With High-risk Disease

Of the 6029 patients with high-risk neuroblastoma, 2473 (41.0%) were enrolled in an up-front clinical trial, and 3556 were enrolled in biology studies only. Similar to both US census data^26^ and cancer prevalence percentages,^28,29^ 316 of 2154 patients with high-risk neuroblastoma in clinical trials (14.7%) and 446 of 3132 of patients with high-risk neuroblastoma in biology studies (14.2%) were Black. Hispanic patients made up 11.7% (586 of 5014) of the group of patients with high-risk disease. Compared with patients enrolled only in biology studies, those enrolled in a clinical trial were more likely to be older than 18 months at diagnosis (2149 [86.9%] vs 2953 [83.0%]; *P* < .001), have INSS stage 4 disease (2151 of 2427 [88.6%] vs 2945 of 3512 [83.9%]; *P* < .001), have unfavorable histologic characteristics (1734 of 1819 [95.3%] vs 2429 of 2610 [93.1%]; *P* = .002), have hypodiploidy or diploidy (806 of 1486 [54.2%] vs 1357 of 2665 [50.9%]; *P* = .04), and have undifferentiated or poorly differentiated tumors (1697 of 1737 [97.7%] vs 2247 of 2347 [95.7%]; *P* = .001). There were no detectable differences in enrollment according to sex, race/ethnicity, *MYCN* amplification status, or MKI.

### Outcomes for High-risk Patients

**Figure 2.**
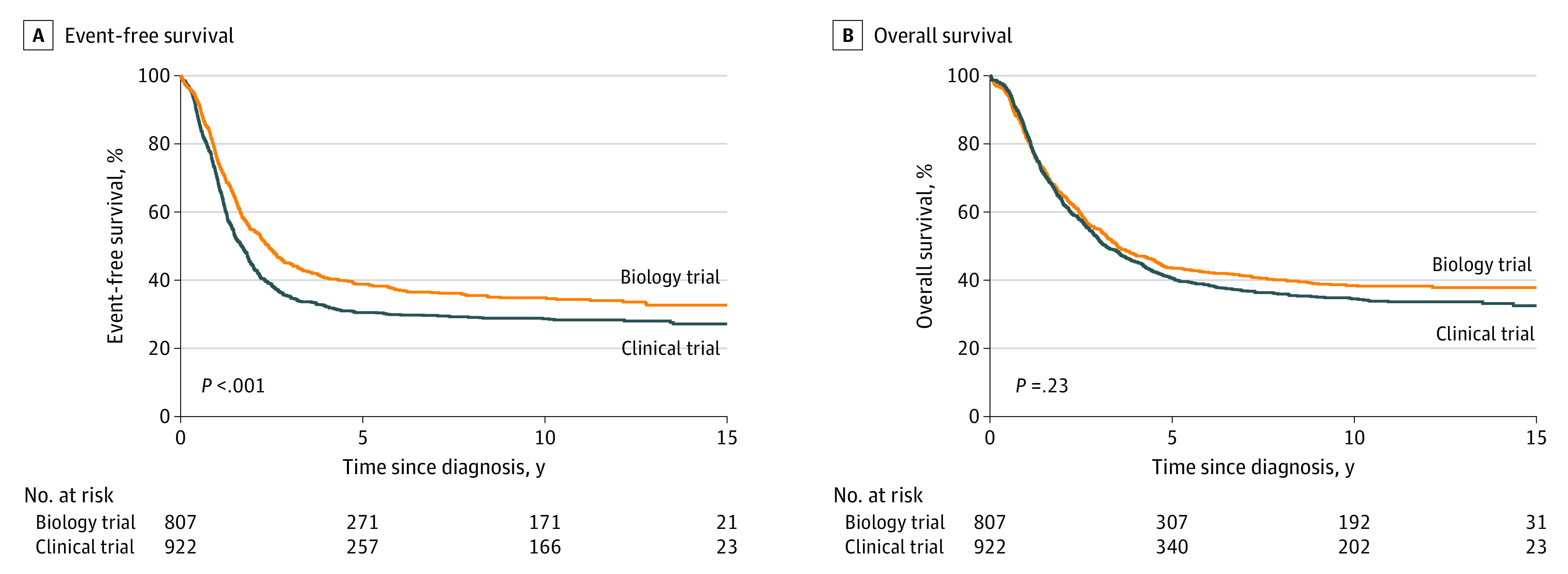
Outcomes for Patients With High-risk Neuroblastoma A, Probability of event-free survival. B, Probability of overall survival. Patients were enrolled in 2 clinical trials (Children’s Cancer Group 3891 or Children’s Oncology Group A3973 [n = 922]) or in a biology study alone (n = 807).

Clinical trial outcomes data for this analysis were limited to patients enrolled in CCG 3891, conducted between 1991 and 1997, and COG A3973, conducted between 2001 and 2006, which enrolled at least 100 patients. A significantly lower EFS was observed for patients who participated in COG A3973 and CCG 3891 (n = 922) compared with those enrolled only in a biology study who received a diagnosis between 1991 and 1997 or between 2001 and 2006 (n = 807) (32% [95% CI, 29%-35%] vs 38% [95% CI, 35%-41%]; *P* < .001 at 10 years (Figure 2A). The median follow-up time of survivors was 11 years (IQR, 7.5-13.4 years) in clinical trials and 10.2 years (IQR, 5.5-12.5 years) in biology studies. In the Cox proportional hazards regression model, clinical trial enrollment remained significantly associated with inferior EFS (HR, 1.16; 95% CI, 1.02-1.33; *P* = .02) compared with biology study enrollment when accounting for stage and *MYCN* status (eTables 1 and 2 in the Supplement). However, no significant difference in OS between the 2 groups (38% [95% CI, 35%-41%] vs 41% [95% CI, 38%-44%]; *P* = .23) (Figure 2B) was observed (Table 2). Similarly, there was no difference in OS according to clinical trial enrollment (HR, 1.01; 95% CI, 0.89-1.16; *P* = .81) when accounting for stage and *MYCN* status (eTables 1 and 2 in the Supplement).

To investigate whether the differences in EFS may be due to a delay in reporting events other than death for patients enrolled in biology studies, we compared the time between the reported event and survival among the patients enrolled in clinical trials and patients enrolled in biology studies only. Among the 807 patients treated in a biology study only, event and death were reported on the same day for 224 of 464 deceased patients (48.3%) compared with 76 of 587 deceased patients (13.0%) enrolled in COG A3973 or CCG 3891 (*P* < .001).

**Figure 3.**
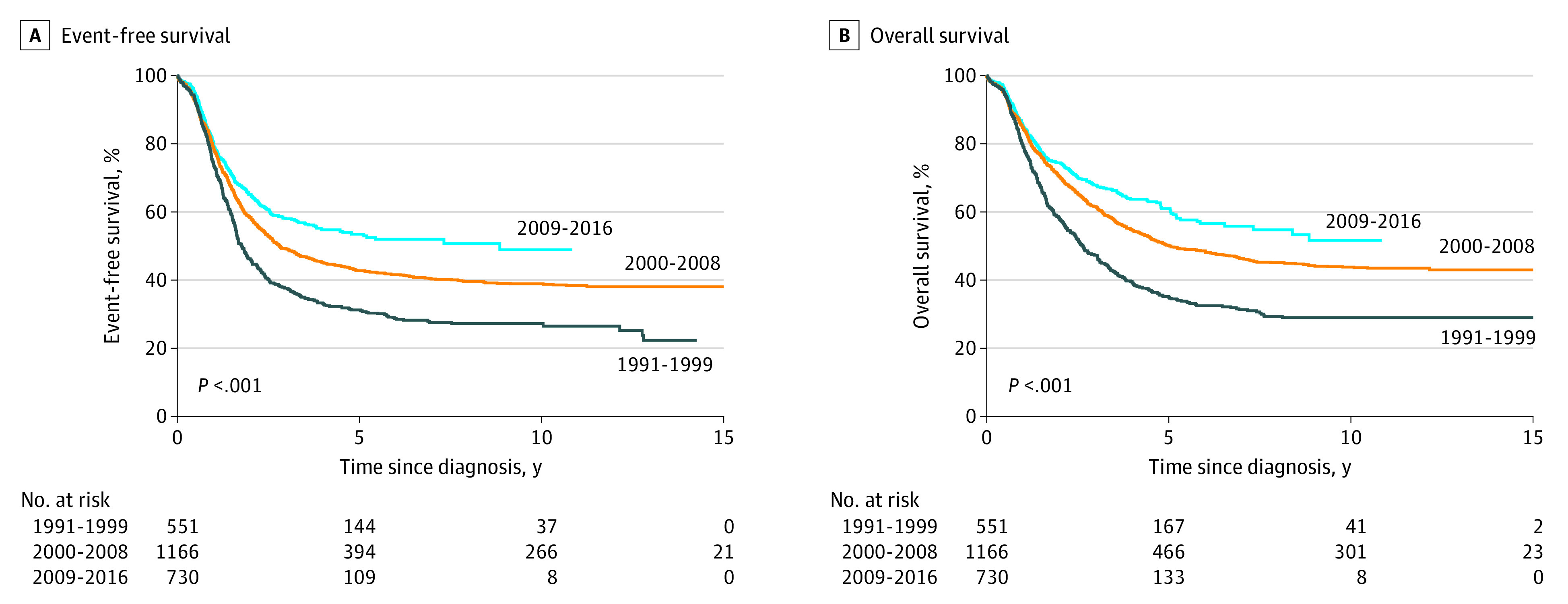
Outcomes for Patients With High-risk Neuroblastoma According to Era in Which Standard of Care Was Changed A, Probability of event-free survival. B, Probability of overall survival. Patients (n = 2447) with high-risk neuroblastoma were enrolled in a biology study alone and received a diagnosis between 1991 and 2016. Standard of care was changed between eras based on the results of a prospective, randomized, cooperative group trial.

To evaluate how outcomes changed over time for patients not enrolled in a clinical trial, we analyzed EFS and OS of 2447 patients with high-risk disease enrolled in a biology study but not an up-front clinical trial according to 3 eras (1991-1999, 2000-2008, and 2009-2016) corresponding to changes in standards of care.^30,31^ Both EFS and OS were superior for patients treated in more recent eras (Figure 3), suggesting that all patients with high-risk disease are experiencing benefits associated with the advances made in clinical trials.

## Discussion

In this study, we analyzed 9087 patients with neuroblastoma in the INRG Data Commons to investigate whether participation in an up-front clinical trial was associated with superior outcomes. Most patients in North America who received a diagnosis of neuroblastoma during the past 3 decades were enrolled in the COG ANBL00B1 or legacy biology study, and demographic information, tumor biomarkers, and outcome data on these patients are included in the INRG Data Commons. A total of 43.9% of the patients with intermediate- or high-risk disease were also enrolled in a clinical trial; for these patients, the number that identifies the clinical trial are captured in the INRG Data Commons. These data provide a unique opportunity to compare the outcomes of North American patients with neuroblastoma enrolled in a clinical trial with a representative cohort of “real-world” patients who were treated off trial.

Because of the advances in neuroblastoma treatment that have been made based on sequential clinical trials testing new therapeutic approaches,^6^ we expected to find a benefit associated with clinical trial participation for patients with high-risk disease. However, our analysis demonstrated that OS was not higher for patients with high-risk disease enrolled in an up-front clinical trial compared with those treated off trial. The reasons for the lack of survival benefit remain unclear but may reflect the common practice to treat patients not enrolled in a clinical trial according to the therapeutic and supportive care regimens used in a previous clinical trial.^32^

We also found that EFS was inferior for patients with high-risk disease who were enrolled in an up-front clinical trial compared with those who were treated off trial. Comparison of the 2 cohorts demonstrated that the patients enrolled in clinical trials had a higher prevalence of high-risk features, including older age, metastatic disease, and unfavorable biological features. These differences in clinical features and tumor biology suggest that there may be physician bias regarding enrollment of patients with high-risk disease in clinical trials. To assess other possible reasons for the improved EFS in the biology study cohort, we evaluated the time from event to death and found that a significantly larger proportion of patients in biology studies had 0 days between event and death compared with those in clinical trials. These findings suggest that the superior EFS observed in the children enrolled only in a biology study may be due, in part, to a failure to report events other than death.

In contrast to the cohort of patients with high-risk disease, significantly improved OS but not EFS was observed for the patients with intermediate-risk disease who were enrolled in up-front clinical trials. This observation suggests that salvage treatments after relapse were more effective in the clinical trial cohort, which may reflect differences in tumor biology. Analysis of the 2 cohorts demonstrated that patients enrolled in an intermediate-risk clinical trial were significantly more likely to have favorable prognostic markers, including localized disease and tumors with favorable biological features. In a multivariable analysis accounting for age, disease stage, and ploidy, enrollment in a clinical trial was not significantly associated with OS, suggesting that differences in these features were associated with the observed difference in OS. Thus, there appears to be physician bias toward off-trial treatment of patients with intermediate-risk disease with more unfavorable tumor biology.

Contrasting studies identifying discrepancies in clinical trial enrollment according to demographic features, such as older age, and race/ethnicity,^28,33,34,35,36,37,38,39,40^ we found no evidence of bias in recruitment across demographic groups. Of the 3986 patients enrolled in studies, 12.9% were Black and 11.3% were Hispanic, mirroring the prevalence of Black and Hispanic individuals in the US population and in the overall neuroblastoma population in North America. Previous studies have shown that Black and Native American children have a higher prevalence of high-risk disease,^41^ and there may be factors genetically predisposing these groups to have more aggressive tumors.^42^ Our study suggests that differences in outcomes are not likely due to whether or not a patient is enrolled in a clinical trial. Although we are unable to assess how other social determinants of health that disproportionally affect minority populations may be associated with adherence to protocol therapy and outcomes,^33^ virtually all chemotherapy regimens for neuroblastoma are administered intravenously in a hospital or outpatient clinic and closely monitored.

### Limitations

This study has some limitations. Information about treatment received is not available in the INRG Data Commons. Although postconsolidation immunotherapy has been shown to improve survival for patients with high-risk neuroblastoma,^43^ outcome data for patients enrolled in the nonrandomized immunotherapy expansion group of the ANBL0032 clinical trial are not currently available in the INRG Data Commons. Specifically, the biology study–only cohort did not include 423 patients who received a diagnosis between 2009 and 2016 who were not enrolled in an up-front therapeutic trial but enrolled in ANBL0032 and nonrandomly assigned to receive postconsolidation immunotherapy. Thus, the actual EFS and OS of the patients enrolled in a biology-only study during this era is likely higher than reported in this study.

## Conclusions

To learn from every pediatric oncology patient, there is a culture among pediatric oncologists to ask every parent or legal guardian to consider enrolling their child in an up-front clinical trial. This INRG Data Commons analysis found that, among patients with intermediate- or high-risk neuroblastoma diagnosed in North America, there was a high prevalence of population-wide clinical trial participation. Advances in neuroblastoma treatment during the past decades have resulted from the development of new standards of care based on the results of successive, risk-based clinical trials, improving survival rates of patients with high-risk disease. Our results suggest that there may be some physician bias regarding clinical trial enrollment associated with tumor biology. However, no evidence of bias in recruitment across demographic groups was observed, enabling assessment of treatment response and toxic effects across racial/ ethnic groups. The decision to enroll in clinical trials can be fraught with tension^18^ but must continue to be supported and encouraged.

## Notes

Supplement. **eTable 1.** Univariate Cox Proportional Hazards Regression Models of Event-Free and Overall Survival for High-risk and Intermediate-risk Patients **eTable 2.** Multivariate Cox Proportional Hazards Regression Models of Event-Free and Overall Survival for High-risk and Intermediate-risk Patients
