# Supplementary material for: Association Between Participation in Clinical Trials and Overall Survival Among Children With Intermediate- or High-risk Neuroblastoma
Source: JAMA Netw Open. 2021 Jul 8;4(7):e2116248. doi: 10.1001/jamanetworkopen.2021.16248 (PMC8267607; doi:10.1001/jamanetworkopen.2021.16248)
Supplement: Supplement. — eTable 1. Univariate Cox Proportional Hazards Regression Models of Event-Free and Overall Survival for High-risk and Intermediate-risk Patients eTable 2. Multivariate Cox Proportional Hazards Regression Models of Event-Free and Overall Survival for High-risk and Intermediate-risk Patients [file jamanetwopen-e2116248-s001.pdf]

Supplementary Online Content

Balyasny S, Lee SM, Desai AV, et al. Association between participation in clinical trials and overall survival among children with intermediate- or high-risk neuroblastoma. *JAMA Netw Open.* 2021;4(7):e2116248.
doi:10.1001/jamanetworkopen.2021.16248

eTable 1. Univariate Cox Proportional Hazards Regression Models of Event-Free and Overall Survival for High-risk and Intermediate-risk Patients
eTable 2. Multivariate Cox Proportional Hazards Regression Models of Event-Free and Overall Survival for High-risk and Intermediate-risk Patients

This supplementary material has been provided by the authors to give readers additional information about their work.

© 2021 Balyasny S et al. *JAMA Network Open.*

eTable 1. Univariate Cox Proportional Hazards Regression Models of Event-Free and Overall Survival for High-risk and Intermediate-risk Patients

	**Factor**	**Hazard Ratio (95% CI)**	***P* value**
EFS high-risk patients			
	Therapeutic trial enrollment	1.24 (1.10-1.39)	<0.001
	MYCN-amplification	1.41 (1.24-1.61)	<0.001
	INSS stage[a]		
	3	2.11 (0.52-8.61)	0.30
	4	4.97 (1.24-19.99)	0.02
	4S	3.28 (0.78-15.18)	0.13
OS high-risk patients			
	Therapeutic trial enrollment	1.07 (0.95-1.21)	0.23
	MYCN-amplification	1.61 (1.40-1.84)	<0.001
	INSS stage[a]		
	3	3.77 (0.52-27.16)	0.19
	4	8.51 (1.19-60.48)	0.03
	4S	6.77 (0.86-53.46)	0.07
EFS intermediate-risk patients			
	Therapeutic trial enrollment	1.36 (0.97-1.92)	0.07
	INSS stage[a]		
	3	0.4 (0.26-0.61)	<0.001
	4	0.71 (0.47-1.07)	0.10
	4S	0.86 (0.54-1.35)	0.50
	Hypodiploid/ diploid	1.63 (1.22-2.16)	0.001
	Unfavorable histology	1.60 (1.01-2.55)	0.04
OS intermediate-risk patients			
	Therapeutic trial enrollment	0.59 (0.41-0.085)	0.004
	Age >18 months	0.51 (0.29-0.88)	0.02
	INSS stage[a]		
	3	1.27 (0.38-4.23)	0.70
	4	3.96 (1.24-12.64)	0.02
	4S	5.04 (1.53-16.57)	0.008
	Hypodiploid/ diploid	2.26 (1.49-3.41)	<0.001

[a] Reference group is INSS stage 2

© 2021 Balyasny S et al. *JAMA Network Open.*

eTable 2. Multivariate Cox Proportional Hazards Regression Models of Event-Free and Overall Survival for High-risk and Intermediate-risk Patients

	**Factor**	**Hazard Ratio (95% CI)**	***P* value**
EFS high-risk patients			
	Therapeutic trial enrollment	1.16 (1.02-1.33)	0.02
	MYCN-amplification	1.5 (1.31-1.7)	<0.001
	INSS stage[a]		
	3	2.66 (0.65-10.86)	0.17
	4	6.01 (1.5-24.1)	0.01
	4S	3.95 (0.85-18.28)	0.08
OS high-risk patients			
	Therapeutic trial enrollment	1.01 (0.89-1.16)	0.81
	MYCN-amplification	1.69 (1.47-1.94)	<0.001
	INSS stage[a]		
	3	5.2 (0.72-37.5)	0.10
	4	11.19 (1.57-79.69)	0.02
	4S	8.21 (1.04-64.86)	0.05
EFS intermediate-risk patients			
	Therapeutic trial enrollment	1.36 (0.97-1.92)	0.07
	INSS stage[a]		
	3	0.9 (0.42-1.91)	0.79
	4	1.53 (0.73-3.21)	0.26
	4S	1.16 (0.48-2.76)	0.75
	Hypodiploid/ diploid	1.57 (1.11-2.2)	0.009
	Unfavorable histology	1.37 (0.81-2.32)	0.24
OS intermediate-risk patients			
	Therapeutic trial enrollment	0.68 (0.45-1.03)	0.07
	Age >18 months	0.87 (0.46-1.63)	0.66
	INSS stage[a]		
	3	1.11 (0.33-3.75)	0.87
	4	2.67 (0.79-9.04)	0.11
	4S	2.14 (0.56-7.85)	0.27
	Hypodiploid/ diploid	2.14 (1.4-3.29)	<0.001

[a] Reference group is INSS stage 2

© 2021 Balyasny S et al. *JAMA Network Open.*
